# Literacy and healthcare-seeking among women with low educational attainment: analysis of cross-sectional data from the 2011 Nepal demographic and health survey

**DOI:** 10.1186/1475-9276-12-95

**Published:** 2013-12-13

**Authors:** Yukyan Lam, Elena T Broaddus, Pamela J Surkan

**Affiliations:** 1 Department of International Health, Social and Behavioral Interventions Program, Johns Hopkins Bloomberg School of Public Health, 615 N. Wolfe Street, Room E5527, Baltimore, MD 21205, USA; 2 Fulbright Student Research Program, United States Education Foundation, Gyaneshwor, GPO Box 380, Kathmandu, Nepal

**Keywords:** Nepal, Literacy, Women, Agency, Healthcare decision-making, Care-seeking, Healthcare access, Social epidemiology, Social determinants of health

## Abstract

**Introduction:**

Research suggests that literacy plays a key role in mediating the relationship between formal education and care-seeking among women in developing countries. However, little research has examined literacy’s role independently from formal education. This differentiation is important, as literacy programs and formal schooling entail distinct intervention designs and resources, and may target different groups. To assess the relationship between literacy and healthcare-seeking among Nepali women of low educational attainment, we analyzed data from the 2011 Nepal Demographic and Health Survey (DHS).

**Methods:**

From the 2011 Nepal DHS, our sample consisted of 7,020 women who had attained at most a primary school level of education, and a subsample of 4,875 women with no formal schooling whatsoever. We assessed associations between literacy and four healthcare-seeking outcomes: whether women identified “getting permission” as a barrier to accessing care; whether women identified “not wanting to go alone” as a barrier; whether among women who were married/partnered, the woman had some say in making decisions about her own health; and whether among women who experienced symptoms related to sexually-transmitted infections (STIs) in the past year, treatment was sought. We performed simple and multiple logistic regressions, which adjusted for several socio-demographic covariates.

**Results:**

Literacy was associated with some aspects of healthcare-seeking, even after adjusting for socio-demographic covariates. Among women with no more than primary schooling, literate women’s odds of identifying “getting permission” as a barrier to healthcare were 23% less than illiterate women’s odds (p = 0.04). For married/partnered women, odds of having some say in making decisions related to their health were 37% higher (p = 0.002) in literate than illiterate women. Comparing literate to illiterate women in the subsample with no formal schooling, odds of reporting “getting permission” as a barrier were 35% lower (p = 0.01), odds of having a decision-making say were 57% higher (p < 0.001), and odds of having sought care for experiences of STI-related symptoms were 86% higher (p = 0.04).

**Conclusions:**

Further research should be undertaken to determine whether targeted literacy programs for those past normal schooling age lead to improved healthcare-seeking among Nepali women with little or no formal education.

## Background

Country-wide estimates from 2011 suggest that two in five Nepali women have never attended school, and a third of women ages 15–49 are illiterate [1]. The proportion of women with no formal education increases with age, and older age is also associated with lower levels of literacy [1]. At the same time that Nepal is attempting to meet the Millennium Development Goals for education, it is also working to improve maternal health and reduce child mortality. Increasing women’s utilization of healthcare services is recognized as important for achieving these health outcomes [1].

The connection between women’s educational attainment and health service utilization is well documented in Nepal and elsewhere in the developing world [2-8]. Yet there is a lack of consensus on which aspects of education most influence health behaviors. Many researchers argue that education alters identity, increasing self-confidence and leading women to form enhanced self-perceptions and to practice new behaviors [9-11]. Others contend that formal education transmits behavioral norms such as openness to “modern” medicine and adherence to the schedules and bureaucratic processes that health systems require [2,12,13]. Increasing evidence suggests that providing literacy skills is the key function of formal education relevant to health outcomes, because these skills allow women to access health information and to more effectively navigate health systems [3,14-19].

Studies examining literacy skills and health behavior have found literacy to be an important predictor of a woman’s likelihood of accessing healthcare for herself or her child [3,18,20]. Previously, many researchers implicitly or explicitly treated literacy and education level as proxies for each other, in spite of their differences. However, studies in Nepal [15,17,18], as well as in Mexico [21], Zambia [22], and Venezuela [23] have aimed to disentangle these differences by measuring multiple types of literacy using a variety of methods, and by controlling for schooling and other socioeconomic factors in their analyses [19]. Findings indicate that even in poor quality schools or with a very small amount of schooling, women often manage to gain some literacy skills and retain these skills into adulthood [19]. These literacy skills are the key mediator through which maternal education impacts the health outcomes of their children [19]. We sought to build on these findings by exploring the impact of literacy as a determinant of health behavior independent of formal education [24,25]. Additionally, rather than examining behaviors related specifically to child health outcomes, we examined behaviors related to women’s care-seeking for their own health. We sought to observe this relationship in the context of Nepal, a developing country with high levels of illiteracy and low educational attainment.

Understanding the impact of literacy on healthcare utilization, independent of formal education, has important implications in Nepal. There are many women with little or no formal education who acquire literacy skills through other channels, such as from family members or through adult literacy programs [1,3]. Evidence that literacy itself – and not *only* formal schooling – improves health may motivate expansion of programs that can benefit adults who are past school age. Thus, to expand the evidence base on this topic, we assessed the association between literacy and several behaviors and barriers related to accessing healthcare. We hypothesized that literacy would be associated with increased care-seeking or capacity for care-seeking among Nepali women with little or no primary schooling.

## Methods

### Study population

We conducted secondary data analysis of the 2011 Nepal Demographic and Health Survey (DHS) data [1,26]. The DHS is a nationally representative survey collected for the purpose of generating data on population and health indicators [1]. The Population Division of the Nepali Ministry of Health and Population oversaw the 2011 DHS, with funding from the United States Agency for International Development (USAID) [1]. The 2011 DHS was the fourth DHS survey conducted in Nepal, and included a sample of 12,674 women and 121 men between the ages of 15 and 49 years old [1].

**Figure 1:**
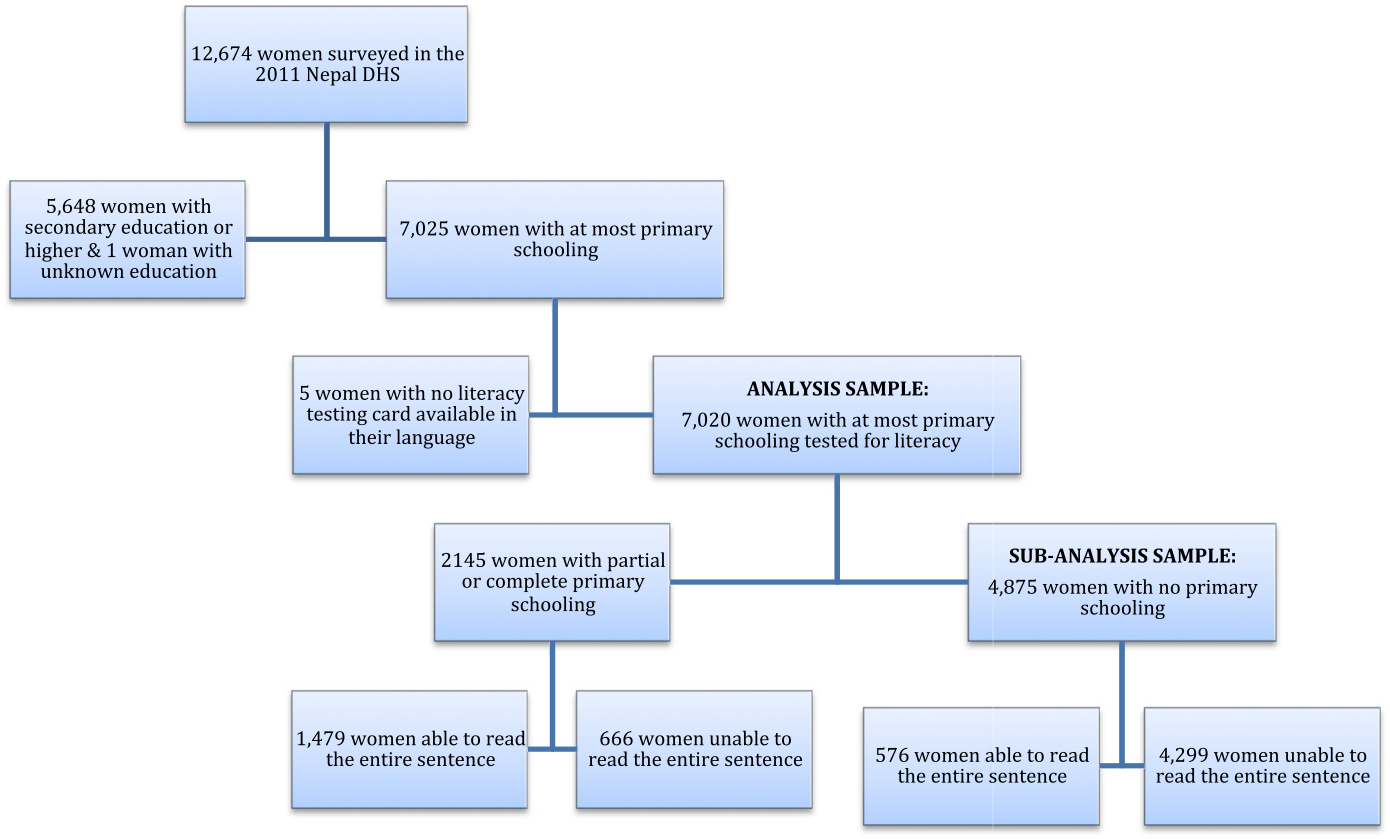
**Sample of surveyed women whose literacy was evaluated.** The sample in our analysis consisted of 7,020 women with no more than primary schooling who had their literacy assessed. The sub-analysis was performed on the 4,875 women who had no formal schooling experience.

Given our focus on the association between literacy and health among women of low educational attainment, we restricted our analysis to a subset of 7,025 women who had received at most a primary school level of education. We excluded women with secondary education or higher because their literacy was not assessed by the surveyor, as they were assumed to be fully literate. Out of the 7,025 women with no more than primary schooling, we excluded five women whose literacy was not assessed because the testing card was not available in their language [1] (See Figure 1). In our final sample of 7,020 women who acquired at most primary schooling and participated in the literacy test, a subsample of 4,875 women had no formal schooling whatsoever.

### Variables

Literacy in the 2011 DHS was tested by asking the respondent to read a sentence on a testing card shown by the surveyor. Thereafter, the surveyor recorded whether the respondent could read the entire sentence, parts of the sentence, or no part of the sentence, and respondents were subsequently categorized as fully literate, partially literate, or illiterate [1]. As our predictor of interest, literacy was treated as binary, with the exposed group including women with low education who could read the entire sentence, and the unexposed group including women with low education who could only read limited parts or no part of the sentence shown [1]. Although treating literacy as a binary variable is a dramatic simplification of what is a continuum of ability [19,27], we made this decision for two reasons. First, we found the “partially literate” category within the original DHS survey to be quite ambiguous. Second, we theorized that it was full basic literacy (as measured by the DHS) that would lead to the improved care-seeking behaviors of interest.

We chose four outcomes to reflect different facets of the concept of care-seeking. Two dichotomous outcomes addressed barriers to healthcare: (i) whether respondents identified “getting permission to go” and (ii) whether respondents identified “not wanting to go alone”, as big problems in getting medical care when sick. A third outcome addressed the issue of healthcare-related agency and decision-making power. Women who were married or living with a partner as if married were asked who usually makes the decisions about healthcare related to the women’s own health. We dichotomized this outcome, distinguishing women who have *no* say in these decisions from those women who have either *complete* or *joint* decision-making power shared with their partner. Finally, the fourth binary outcome measured actual healthcare-seeking. Among women who had experienced a sexually-transmitted infection (STI) or symptoms associated with STIs (discharge or genital sore/ulcer) within the past year, we evaluated whether or not they sought advice or treatment for the problem.

Regarding the socio-demographic covariates, formal schooling was modeled as a nominal categorical variable, indicating whether women had no formal schooling whatsoever, incomplete primary schooling, or complete primary schooling. Age was modeled as a two-piece linear spline, with a knot at 35 years of age. Hence, there were two different regression coefficients, one for women below 35 years of age and the other for women 35 and older. We introduced a breakpoint at 35 years to address non-linearity in the relationship between log odds of the outcomes with age, which was revealed by lowess plots. Wealth was modeled as an ordinal variable, using category scoring (0–4) to designate the wealth quintile of the woman’s household (0 = lowest, 4 = highest). Lowess plots revealed sufficient linearity to permit category scoring—i.e., the use of a single regression coefficient to represent the increase in log odds of the outcomes, from one wealth quintile to the next. Caste/ethnicity was treated as a categorical variable, with the following four groups: (i) Hill Brahmin, Hill Chhetri, Terai Brahmin, and Terai Chhetri; (ii) Newar, Hill Janajati and Terai Janajati; (iii) Hill Dalit and Terai Dalit; and (iv) Other, which included other Terai caste, Muslim and others. Geographic setting was dichotomized as rural or urban. Partnered status was also included as a binary variable, distinguishing women who were married or living with a partner, from women who were widowed, divorced, separated, or had never been in a union.

### Statistical analysis

Data were analyzed as survey data, using STATA statistical software, version 12 (Stata Corp, College Station TX) [28,29]. The analyses described were conducted first for the entire sample of 7,020 women with low educational attainment, and then for the subsample of 4,875 women with no formal schooling whatsoever. For each of the four binary care-seeking outcomes, we performed simple logistic regressions to assess the unadjusted effects of literacy and each of the socio-demographic variables. Thereafter, we performed multiple logistic regressions to assess the effect of literacy after adjusting for age, wealth, caste/ethnicity, geographic setting, and partnered status as potential confounders. In the multiple logistic regression model for the decision-making power outcome, we did not incorporate partnered status as a covariate because the survey only assessed this outcome for women with partners. The multiple logistic regressions for the broader sample of 7,020 women also included the trichotomous covariate of primary schooling.

Multicollinearity among the variables included in the multiple logistic regression analyses was assessed by performing multiple regression analyses, weighted to account for the survey nature of our data [26,29], to calculate variance inflation factors (VIFs). Mean VIFs across the four outcomes ranged from 1.81 to 1.88 for the sample, and from 1.87 to 2.00 for the subsample, indicating minimal multicollinearity. An F-adjusted mean residual test [29] developed by Archer and Lemeshow [30] was used to assess goodness-of-fit of the survey design-based logistic regression models. The test indicated that the models were a good fit for our survey data, as p-values for the four outcomes ranged from 0.05 to 0.90 for the sample, and from 0.47 to 0.92 for the subsample.

The Institutional Review Board of the Johns Hopkins Bloomberg School of Public Health determined that this research did not qualify as human subjects research as defined by DHHS regulations 45 CFR 46.102, as it was considered secondary data analysis of an existing, de-identified and publicly available dataset. In accordance with this determination, the IRB deemed the research exempt from oversight.

## Results

In our sample of 7,020 Nepali women with at most primary schooling, 4,965 women were literate, and 2,055 women were illiterate, corresponding to survey-weighted proportions of 72.3% and 27.7%, respectively. Socioeconomic and other demographic characteristics for these two groups are shown in Table 1.

**Table 1:** Characteristics by literacy group, among Nepali women with low educational attainment

	**Total (n = 7,020)**	**Literate group (n = 2,055)**	**Illiterate group (n = 4,965)**	***P *** **value**^**a**^
**Age in years, mean (SE**^**b**^**)**	32.32 (0.27)	29.84 (0.38)	33.27 (0.36)	< 0.0001
**Primary school, %**				< 0.0001
None	69.6	26.3	86.1	
Incomplete	20.9	42.3	12.6	
Complete	9.6	31.4	1.2	
**Marital status, %**				< 0.0001
Never in a union	7.5	11.8	5.9	
Married	88.6	85.4	89.8	
Widowed	2.9	2.0	3.2	
Divorced	< 0.1	< 0.1	0.1	
Separated	0.9	0.8	1.0	
**Wealth quintile**^**c**^**, %**				< 0.0001
First	24.4	18.6	26.7	
Second	24.2	21.9	25.1	
Third	23.2	21.3	23.9	
Fourth	18.3	22.5	16.6	
Fifth	9.9	15.7	7.7	
**Geographic location, %**				0.006
Rural	90.9	88.7	91.8	
Urban	9.1	11.3	8.2	
**Caste/Ethnicity, %**				< 0.0001
Brahmin or Chhetri	25.6	31.6	23.3	
Newar or Janajati	39.3	49.3	35.4	
Dalit	19.2	14.3	21.0	
Muslim or other	15.9	4.8	20.2	

^a^*P* value for continuous variable (age) was calculated from an adjusted Wald test comparing mean age of the two groups. *P* values comparing proportions in the two groups are Pearson, survey design-corrected *p* values.

^b^Standard error is linearized to account for survey design.

^c^Cut-off points for the household wealth quintiles were calculated from the 10,826 households surveyed in the 2011 Nepal DHS.

Comparisons showed statistically significant differences between literate and illiterate women in all of the socio-economic and demographic characteristics examined. Literate women were younger (mean = 29.8 years, SD = 0.4 years) compared to illiterate women (mean = 33.3 years, SD = 0.4 years). Literate women were also more likely to have attended formal schooling compared to illiterate women (for example, only 1.2% of illiterate women had completed primary school, compared to 31.4% of literate women). Literate women’s households tended to be better off compared to those of illiterate women (15.7% versus 7.7% of households were in the wealthiest quintile, for example). High proportions of women in both groups were married (85.4% in the literate group; 89.8% in the illiterate group) and lived in a rural area (88.7% in the literate group; 91.8% in the illiterate group).

Among our subsample of 4,875 women with no formal schooling whatsoever, 4,299 were illiterate and 576 were literate, corresponding to survey-weighted proportions of 89.5% and 10.5%, respectively. The subsample showed no statistically significant differences in average age or marital status between the illiterate and literate groups. However, as with the broader sample, illiterate women in our subsample tended to live in poorer households. High proportions of illiterate and literate women in our subsample were married and resided in rural areas (See Table 2).

**Table 2:** Characteristics by literacy group, among Nepali women with no formal schooling

	**Total (n = 4,875)**	**Literate group (n = 576)**	**Illiterate group (n = 4,299)**	***P *** **value**^**a**^
**Age in years, mean (SE**^**b**^**)**	34.22 (0.36)	35.05 (0.50)	34.13 (0.38)	0.117
**Marital status, %**				0.412
Never in a union	4.6	5.4	4.6	
Married	90.8	90.2	90.8	
Widowed	3.5	4.2	3.5	
Divorced	0.1	0.0	0.1	
Separated	0.9	0.3	1.0	
**Wealth quintile**^**c**^**, %**				< 0.0001
First	26.9	17.7	27.9	
Second	25.9	22.8	26.3	
Third	23.1	22.0	23.2	
Fourth	16.6	23.7	15.7	
Fifth	7.6	13.8	6.9	
**Geographic location, %**				0.020
Rural	92.1	89.0	92.5	
Urban	7.9	11.0	7.5	
**Caste/Ethnicity, %**				< 0.0001
Brahmin or Chhetri	23.8	30.2	23.0	
Newar or Janajati	38.3	57.8	36.0	
Dalit	19.7	9.2	20.9	
Muslim or other	18.3	2.8	20.1	

^a^*P* value for continuous variable (age) was calculated from an adjusted Wald test comparing mean age of the two groups. *P* values comparing proportions in the two groups are Pearson, survey design-corrected *p* values.

^b^Standard error is linearized to account for survey design.

^c^Cut-off points for the household wealth quintiles were calculated from the 10,826 households surveyed in the 2011 Nepal DHS.

Table 3 shows the proportion of women in each group who experienced the four outcomes. Among our sample of 7,020 women of low educational attainment, 16.6% stated that obtaining permission to go was a big impediment to accessing healthcare when needed. Moreover, 67.7% of the women identified that not wanting to go alone was a big problem in accessing healthcare. Proportions of women perceiving these barriers were higher among illiterate women compared to literate women. In addition, 62.6% of the 6,232 married/partnered women reported having some say in making decisions related to their own health. This proportion was higher among literate women compared to illiterate women (67.7% versus 60.8%, p < 0.001). Finally, 43.8% of the 845 women who experienced STIs or STI-related symptoms in the past year sought care for these problems. A higher proportion of women in the literate group compared to illiterate group sought care (54.1% versus 39.8%, p = 0.001).

**Table 3:** Percent of women with low educational attainment, by literacy group, who experienced each healthcare-seeking barrier

**Outcome**	**Total N**	**Percent of total**	**Literate group**	**Illiterate group**	***P *** **value**^**a**^
**Perceived “getting permission” to be a big problem**	7,020	16.6%	14.5%	17.4%	0.041
**Perceived “not wanting to go alone” to be a big problem**	7,020	67.7%	62.9%	69.6%	< 0.001
**Had a say in decision-making regarding one’s own health**	6,232	62.6%	67.7%	60.8%	< 0.001
**Sought care for STI/STI symptoms**	845	43.8%	54.1%	39.8%	0.001

^a^*P* values comparing proportions in the two groups are Pearson, survey design-corrected *p* values.

Table 4 provides these same estimates for our subsample of women with no formal schooling. All outcomes, excepting the barrier of not wanting to go alone, were significantly different between literate and literate women: 38.7% of illiterate women versus 61.2% of literate women sought care for STI-related symptoms (p = 0.003), 61.0% of illiterate women versus 74.0% of literate women had a say in decision-making about their health (p < 0.0001), and 17.5% of illiterate women versus 12.0% of literate women perceived getting permission to be a barrier to accessing care (p = 0.006).

**Table 4:** Percent of women with no formal schooling, by literacy group, who experienced each healthcare-seeking barrier

**Outcome**	**Total N**	**Percent of total**	**Literate group**	**Illiterate group**	***P *** **value**^**a**^
**Perceived “getting permission” to be a big problem**	4,875	16.9%	12.0%	17.5%	0.006
**Perceived “not wanting to go alone” to be a big problem**	4,875	69.3%	66.0%	69.7%	0.159
**Had a say in decision-making regarding one’s own health**	4,444	62.4%	74.0%	61.0%	< 0.0001
**Sought care for STI/STI symptoms**	597	41.3%	61.2%	38.7%	0.003

^a^*P* values comparing proportions in the two groups are Pearson, survey design-corrected *p* values.

Table 5 shows the results from the crude and multiple logistic regressions for the first outcome, perceiving “getting permission to go” to be a big barrier in accessing healthcare for oneself when needed. In the unadjusted model, for women with no more than primary schooling who were literate, the odds of perceiving “getting permission to go” to be a barrier were 0.81 (95% CI: 0.66, 0.99) times the odds of that among illiterate women (p = 0.04). After adjusting for primary schooling, age, household wealth, caste/ethnicity, geographic location and partnered status, the odds of identifying getting permission to be a problem was 0.77 times (95% CI: 0.60, 0.99) in literate women compared to illiterate women (p = 0.04). Thus, being literate was associated with an approximate 20% reduction of odds of identifying this barrier among women with at most primary schooling. In our subsample of women with no formal schooling, odds of identifying this barrier were about 35% lower in literate women compared to illiterate women, for both the unadjusted and adjusted models (p = 0.006 and p = 0.012, respectively).

**Table 5:** Crude and adjusted relative odds of perceiving “getting permission to go” to be a problem in accessing healthcare

	**Women with at most primary schooling (sample N = 7,020)**				**Women with no formal schooling (subsample N = 4,875)**			
	**Crude**		**Adjusted**^**a**^		**Crude**		**Adjusted**^**b**^	
	**OR (95% CI)**	***p***^**c**^	**OR (95% CI)**	***p***^**c**^	**OR (95% CI)**	***p***^**c**^	**OR (95% CI)**	***p***^**c**^
**Literacy**		0.042		0.041		0.006		0.012
Illiterate^d^	1.00		1.00		1.00		1.00	
Literate	0.81 (0.66-0.99)		0.77 (0.60-0.99)		0.64 (0.47-0.88)		0.65 (0.46-0.91)	
**Primary schooling**		0.667		0.592				
None^d^	1.00		1.00					
Incomplete	0.90 (0.72-1.13)		0.88 (0.68-1.14)					
Complete	0.96 (0.72-1.26)		0.97 (0.69-1.38)					
**Women’s age (per year)**								
< 35	0.96 (0.95-0.97)	<0.001	0.95 (0.93-0.97)	<0.001	0.96 (0.94-0.98)	0.001	0.96 (0.94-0.98)	<0.001
≥ 35	1.00 (0.97-1.02)	0.828	1.00 (0.98-1.03)	0.800	0.99 (0.97-1.02)	0.574	1.00 (0.97-1.03)	0.933
**Household wealth**								
Per quintile	0.83 (0.76-0.90)	<0.001	0.84 (0.77-0.92)	<0.001	0.86 (0.78-0.95)	0.004	0.87 (0.79-0.96)	0.008
(lowest is ref)								
**Caste/ethnicity**		0.085		0.054		0.242		0.054
Brahmin/Chhetri^d^	1.00		1.00		1.00		1.00	
Newar or Janajati	1.44 (1.09-1.91)		1.36 (1.04-1.79)		1.38 (1.00-1.91)		1.40 (1.02-1.92)	
Dalit	1.20 (0.91-1.58)		0.92 (0.68-1.25)		1.09 (0.79-1.51)		0.89 (0.62-1.28)	
Muslim or other	1.32 (0.85-2.04)		1.07 (0.69-1.66)		1.21 (0.75-1.95)		1.02 (0.62-1.66)	
**Geographic location**		0.818		0.113		0.732		0.325
Urban^d^	1.00		1.00		1.00		1.00	
Rural	1.04 (0.77-1.39)		0.76 (0.54-1.07)		1.06 (0.76-1.48)		0.83 (0.58-1.20)	
**Partnered status**		0.677		0.030		0.638		0.117
Not partnered	1.00		1.00		1.00		1.00	
Married/living with partner	0.95 (0.75-1.21)		1.34 (1.03-1.74)		1.10 (0.75-1.61)		1.35 (0.93-1.98)	

^a^The constant from the multiple logistic regression was -1.707, and the *p*-value from the Archer and Lemeshow goodness-of-fit test for this model was 0.278.

^b^The constant from the multiple logistic regression was -1.777, and the *p*-value from the Archer and Lemeshow goodness-of-fit test for this model was 0.670.

^c^Logistic regression *p* values are adjusted Wald *p* values.

^d^Denotes reference group.

Table 6 shows the results from the crude and multiple logistic regressions for the second outcome, perceiving “not wanting to go alone” to be a big problem in accessing healthcare for oneself when needed. In the unadjusted model for our sample of 7,020 women, the odds of perceiving “not wanting to go alone” to be a barrier among literate women were 0.74 times (95% CI: 0.63, 0.87) the odds of perceiving that barrier among illiterate women (p < 0.001). However, after adjustment for socio-demographic covariates including primary schooling, literacy was no longer a statistically significant predictor of identifying that barrier (p = 0.10). In our subsample of women with no formal schooling, neither the unadjusted nor adjusted models revealed a statistically significant association between literacy and this outcome at the α = 0.05 level.

**Table 6:** Crude and adjusted relative odds of perceiving “not wanting to go alone” to be a problem in accessing healthcare

	**Women with at most primary schooling (sample N = 7,020)**				**Women with no formal schooling (subsample N = 4,875)**			
	**Crude**		**Adjusted**^**a**^		**Crude**		**Adjusted**^**b**^	
	**OR (95% CI)**	***p***^**c**^	**OR (95% CI)**	***p***^**c**^	**OR (95% CI)**	***p***^**c**^	**OR (95% CI)**	***p***^**c**^
**Literacy**		<0.001		0.103		0.159		0.769
Illiterate^d^	1.00		1.00		1.00		1.00	
Literate	0.74 (0.63-0.87)		0.85 (0.71-1.03)		0.84 (0.66-1.07)		0.96 (0.75-1.24)	
**Primary schooling**		0.005		0.048				
None^d^	1.00		1.00					
Incomplete	0.81 (0.67-0.97)		0.83 (0.68-1.01)					
Complete	0.75 (0.61-0.91)		0.77 (0.62-0.96)					
**Women’s age (per year)**								
< 35	0.95 (0.94-0.96)	<0.001	0.95 (0.94-0.96)	<0.001	0.94 (0.93-0.96)	<0.001	0.95 (0.93-0.97)	<0.001
≥ 35	1.03 (1.01-1.04)	<0.001	1.03 (1.01-1.05)	<0.001	1.03 (1.01-1.05)	0.001	1.04 (1.02-1.05)	<0.001
**Household wealth**		<0.001		<0.001		<0.001		<0.001
Per quintile	0.76 (0.71-0.81)		0.81 (0.75-0.87)		0.78 (0.72-0.84)		0.81 (0.75-0.88)	
(Lowest is ref)								
**Caste/ethnicity**		0.114		0.711		0.680		0.958
Brahmin/Chhetri^d^	1.00		1.00		1.00		1.00	
Newar or Janajati	1.20 (0.96-1.49)		1.13 (0.91-1.39)		1.02 (0.78-1.34)		1.06 (0.82-1.37)	
Dalit	1.36 (1.05-1.75)		1.04 (0.81-1.34)		1.18 (0.87-1.61)		1.02 (0.75-1.41)	
Muslim or other	1.19 (0.85-1.67)		1.02 (0.74-1.40)		1.02 (0.69-1.50)		0.97 (0.68-1.41)	
**Geographic location**		<0.001		0.080		<0.001		0.022
Urban^d^	1.00		1.00		1.00		1.00	
Rural	1.78 (1.43-2.22)		1.25 (0.97-1.60)		1.89 (1.48-2.40)		1.37 (1.05-1.79)	
**Partnered status**		<0.001		0.226		0.001		0.100
Not partnered	1.00		1.00		1.00		1.00	
Married/living with partner	0.67 (0.54-0.83)		0.87 (0.69-1.09)		0.65 (0.49-0.84)		0.78 (0.58-1.05)	

^a^The constant from the multiple logistic regression was 0.749, and the *p*-value from the Archer and Lemeshow goodness-of-fit test for this model was 0.178.

^b^The constant from the multiple logistic regression was 0.760, and the *p*-value from the Archer and Lemeshow goodness-of-fit test for this model was 0.917.

^c^Logistic regression *p* values are adjusted Wald *p* values.

^d^Denotes reference group.

Table 7 shows the results from the crude and multiple logistic regressions for having some say (either complete or shared) in making decisions about one’s own health, among women who were married or living with a partner. In the unadjusted model for our total sample, the odds of having some decision-making power among literate women were 1.35 times (95% CI: 1.15, 1.60) that of illiterate women (p < 0.001). After adjusting for socio-demographic covariates, including primary schooling, the odds ratio of having some decision-making power, comparing literate to illiterate women, was 1.37 (95% CI: 1.13, 1.66; p = 0.002). For our subsample of women with no formal schooling, the unadjusted and adjusted models revealed an even stronger association between literacy and the outcome: odds were 81% higher in literate women versus illiterate women in the unadjusted model (p < 0.001), and 57% higher comparing literate to illiterate women in the adjusted model (p < 0.001).

**Table 7:** Crude and adjusted relative odds of having power in making decisions about one’s own health

	**Women with at most primary schooling (sample N = 6,232)**				**Women with no formal schooling (subsample N = 4,444)**			
	**Crude**		**Adjusted**^**a**^		**Crude**		**Adjusted**^**b**^	
	**OR (95% CI)**	***p***^**c**^	**OR (95% CI)**	***p***^**c**^	**OR (95% CI)**	***p***^**c**^	**OR (95% CI)**	***p***^**c**^
**Literacy**		<0.001		0.002		<0.001		<0.001
Illiterate^d^	1.00		1.00		1.00		1.00	
Literate	1.35 (1.15-1.60)		1.37 (1.13-1.66)		1.81 (1.44-2.29)		1.57 (1.23-2.01)	
**Primary schooling**		0.770		0.249				
None^d^	1.00		1.00					
Incomplete	1.06 (0.89-1.27)		1.18 (0.96-1.46)					
Complete	0.98 (0.77-1.24)		1.02 (0.75-1.39)					
**Women’s age (per year)**								
< 35	1.12 (1.10-1.14)	<0.001	1.12 (1.10-1.14)	<0.001	1.13 (1.11-1.15)	<0.001	1.13 (1.10-1.15)	<0.001
≥ 35	0.96 (0.94-0.98)	<0.001	0.96 (0.94-0.98)	<0.001	0.97 (0.95-0.98)	<0.001	0.96 (0.95-0.98)	<0.001
**Household wealth**								
Per quintile	1.10 (1.03-1.17)	0.004	1.05 (0.97-1.13)	0.253	1.09 (1.01-1.18)	0.025	1.06 (0.97-1.15)	0.213
(lowest is ref)								
**Caste/ethnicity**		<0.0001		0.001		<0.001		0.068
Brahmin/Chhetri^d^	1.00		1.00		1.00		1.00	
Newar or Janajati	0.99 (0.80-1.24)		1.10 (0.89-1.35)		1.04 (0.81-1.34)		1.05 (0.82-1.33)	
Dalit	0.80 (0.63-1.02)		1.14 (0.92-1.42)		0.85 (0.64-1.13)		1.13 (0.87-1.47)	
Muslim or other	0.47 (0.36-0.63)		0.66 (0.49-0.88)		0.55 (0.40-0.76)		0.73 (0.52-1.02)	
**Geographic location**		0.058		0.415		0.162		0.724
Urban^d^	1.00		1.00		1.00		1.00	
Rural	0.82 (0.66-1.01)		0.92 (0.74-1.13)		0.83 (0.64-1.08)		0.96 (0.74-1.23)	

^a^The constant from the multiple logistic regression was 1.100, and the *p*-value from the Archer and Lemeshow goodness-of-fit test for this model was 0.054.

^b^The constant from the multiple logistic regression was 1.034, and the *p*-value from the Archer and Lemeshow goodness-of-fit test for this model was 0.590.

^c^Logistic regression *p* values are adjusted Wald *p* values.

^d^Denotes reference group.

Finally, Table 8 shows the results from the simple and multiple logistic regressions for the last outcome, care-seeking for an STI or STI-related symptoms among women who experienced them within the past 12 months. In the simple unadjusted model for women with at most primary schooling, the odds of having sought care among literate women were 1.78 times (95% CI: 1.26, 2.52) those of illiterate women (p = 0.001). However, after adjustment for socio-demographic covariates including primary schooling, literacy was no longer a statistically significant predictor of care-seeking. The odds of having sought care for an STI or STI-related symptoms among literate women were 1.34 times (95% CI: 0.84, 2.15) the odds among illiterate women (p = 0.22). However, in our subsample of women with no formal schooling, the associations were statistically significant, with odds ratios of having sought care comparing illiterate to literate women of 2.49 (p = 0.003) and 1.86 (p = 0.038), for the unadjusted and adjusted models, respectively.

**Table 8:** Crude and adjusted relative odds of having sought care for STI/STI symptoms among women with STI/STI symptoms in the past 12 months

	**Women with at most primary schooling (sample N = 845)**				**Women with no formal schooling (subsample N = 597)**			
	**Crude**		**Adjusted**^**a**^		**Crude**		**Adjusted**^**b**^	
	**OR (95% CI)**	***p***^**c**^	**OR (95% CI)**	***p***^**c**^	**OR (95% CI)**	***p***^**c**^	**OR (95% CI)**	***p***^**c**^
**Literacy**		0.001		0.219		0.003		0.038
Illiterate^d^	1.00		1.00		1.00		1.00	
Literate	1.78 (1.26-2.52)		1.34 (0.84-2.15)		2.49 (1.36-4.56)		1.86 (1.03-3.34)	
**Primary schooling**		0.102		0.969				
None^d^	1.00		1.00					
Incomplete	1.40 (0.93-2.10)		1.07 (0.63-1.82)					
Complete	1.52 (0.83-2.77)		1.05 (0.49-2.23)					
**Women’s age (per year)**								
< 35	1.06 (1.02-1.10)	0.006	1.06 (1.01-1.11)	0.011	1.05 (0.99-1.11)	0.110	1.04 (0.97-1.10)	0.254
≥ 35	0.94 (0.89-1.00)	0.047	0.93 (0.88-0.99)	0.025	0.95 (0.89-1.01)	0.122	0.93 (0.87-1.00)	0.039
**Household wealth**								
Per quintile	1.41 (1.22-1.63)	<0.001	1.42 (1.21-1.66)	<0.001	1.42 (1.19-1.71)	<0.001	1.45 (1.20-1.76)	<0.001
(lowest is ref)								
**Caste/ethnicity**		0.037		0.043		0.101		0.118
Brahmin/Chhetri^d^	1.00		1.00		1.00		1.00	
Newar or Janajati	1.31 (0.87-1.97)		1.23 (0.81-1.88)		1.32 (0.80-2.19)		1.09 (0.65-1.82)	
Dalit	1.28 (0.87-1.87)		1.49 (0.96-2.30)		1.31 (0.84-2.03)		1.33 (0.81-2.18)	
Muslim or other	0.56 (0.31-1.02)		0.62 (0.33-1.17)		0.48 (0.21-1.12)		0.44 (0.18-1.07)	
**Geographic location**		0.089		0.600		0.006		0.435
Urban^d^	1.00		1.00		1.00		1.00	
Rural	0.70 (0.46-1.06)		1.14 (0.69-1.90)		0.50 (0.31-0.82)		0.78 (0.42-1.45)	
**Partnered status**		0.535		0.637		0.718		0.597
Not partnered	1.00		1.00		1.00		1.00	
Married/living with partner	1.32 (0.54-3.24)		1.23 (0.52-2.93)		1.20 (0.44-3.31)		1.30 (0.49-3.48)	

^a^The constant from the multiple logistic regression was -0.921, and the *p*-value from the Archer and Lemeshow goodness-of-fit test for this model was 0.900.

^b^The constant from the multiple logistic regression was -0.640, and the *p*-value from the Archer and Lemeshow goodness-of-fit test for this model was 0.466.

^c^Logistic regression *p* values are adjusted Wald *p* values.

^d^Denotes reference group.

## Discussion

We hypothesized that literacy would be associated with increased care-seeking or capacity for care-seeking among Nepali women of low educational attainment (i.e., women with no more than a primary school level of education). The foregoing analysis revealed that, among these women, literacy was indeed associated with an increase in odds of possessing health-related decision-making power, as well as a decrease in odds of identifying “getting permission to go” to be a barrier in accessing healthcare when needed. These associations remained significant even when accounting for primary school attainment, as well as women’s age, partnered status, geographic location, caste/ethnicity, and household wealth. Notably, for these two outcomes, literacy was a significant predictor in our adjusted and unadjusted models, while exposure to primary schooling was not. At the same time, although literacy was also associated with an increase in odds of care-seeking for STIs and with a reduction in odds of identifying “not wanting to go alone” to be a barrier in accessing healthcare, these associations were not statistically significant in the larger sample after adjustment for socioeconomic and demographic characteristics.

When we repeated the analysis using the subgroup of women with no exposure to formal education whatsoever, the positive association between literacy and health-related decision-making power and the negative association between literacy and identifying permission as a barrier were both strengthened. Also, within this subsample there was a significant positive association between literacy and care-seeking for STIs that was not observed in the larger sample.

Interestingly, for our total sample, the covariate of formal schooling was not statistically significantly associated with three of the four outcomes in either the unadjusted or adjusted models. For the outcome of “not wanting to go alone,” more exposure to formal schooling was significantly associated with reduced odds of identifying that barrier in both the unadjusted and adjusted models. Incidentally, this was the only outcome that was not significantly associated with literacy among those with no formal education. This suggests that there might be some route separate from basic literacy through which formal education impacts the likelihood of identifying “not wanting to go alone” as a barrier.

We are hesitant to over-interpret these findings given the limitation of the DHS’s literacy assessment method discussed below, and because women who manage to become literate without formal schooling may differ from others in ways that we are unable to control for. However, our results are consistent with the hypothesis that literacy has an effect on healthcare-seeking that is independent of formal schooling. Taken together, regression analyses for the four outcomes suggest that literacy is indeed associated with better healthcare-seeking, and that this association is most significant for the dimensions of care-seeking related to women’s power and agency. These findings build upon those of Acharya et al., whose analysis of the Nepal DHS data from 2006 indicated that educational attainment was a key determinant of women’s autonomy in healthcare decision-making. Using Nepal DHS data from 2006, Acharya and colleagues identified how socio-demographic factors influenced women’s ability to make decisions about their own healthcare, as well as other household decisions. They found that higher educational levels—categorized as none, primary, some secondary, and School Leaving Certificate (SLC) and above—were predictive of an increased likelihood that a woman rather than her husband or partner made her own decisions about her healthcare. Literacy, however, was not included as a variable in their model [10].

Our findings also build upon the results of cross-sectional studies by LeVine et al. and Rowe et. al. [15,18]. LeVine and colleagues directly assessed female literacy in 167 Nepali women and found that literacy skills acquired through schooling were correlated with amount of schooling (Person’s *r* ranged from 0.66 to 0.79), and either partially or nearly completely mediated the effect of schooling on improved comprehension of health messages in the media, understanding of medical instructions, and ability to tell a coherent health-related narrative [18]. Rowe and colleagues obtained similar results in their analysis of data from the much larger-scale UNICEF Nepal Literacy and Health Survey, finding that literacy in combination with media exposure explained much of the variation in maternal health knowledge and behavior [15]. Given that it is fairly well established that literacy is a mediator of the relationship between formal schooling and maternal behaviors and knowledge that impact child health outcomes [15,19], here we have sought to extend this work by assessing literacy’s relationship with women’s care-seeking independent of formal schooling, by adjusting for level of education in our regression models and then by conducting a sub-analysis of those women in our sample with no formal education.

Our results suggest that among women with less than secondary school education, acquisition of literacy skills may increase their autonomy in healthcare decision-making, even among those with no formal education. While further analysis is required to confirm our findings, our results concur with the findings of Sandiford et al. in Nicaragua [25] and Govindasamy and Ramesh in India [20], both of whose analyses indicated that among mothers with little or no exposure to formal education, being literate conferred significant benefits related to child health outcomes. Again, our study examined women’s healthcare-seeking practices rather than child health outcomes; however, the mechanisms through which literacy confers benefits may be related.

While additional research is needed to delineate exactly what these mechanisms are, our finding that literacy is significantly associated with dimensions of care-seeking related to power and agency supports the idea presented by Robinson-Pant, based on ethnographic research of an adult women’s literacy program, that acquiring literacy skills brings about altered perceptions of self-identity and improved self-confidence [31]. Some researchers have argued that these are the primary pathways through which formal education impacts health behavior [11]; however, our findings suggest that literacy acquired outside of formal education may lead to many of the same benefits.

Although we found that literacy was related to a number of healthcare behaviors in women with little or no education, our research is not meant to imply that formal primary schooling is not necessary for facilitating care-seeking. The fact that we found formal schooling to be highly associated with literacy itself (e.g., Table 1) reflects this. Also, we were restricted by the available data in the spectrum of care-seeking outcomes we could include, and moreover our results regarding the outcome of identifying “not wanting to go alone” as a barrier suggest that some aspect of primary schooling other than literacy reduces the likelihood of this barrier. Lastly and more broadly, primary school education is obviously important for countless reasons beyond the narrow focus of this study.

It is worth acknowledging that because this was a cross-sectional study, the nature of the data prevents us from claiming causality. However, it is likely that literacy is an exposure usually acquired over the course of many years, which therefore probably precedes the outcomes we chose (perceived barriers, current decision-making power, and STI care-seeking in the past year). In addition, although we observed an association between literacy and healthcare-seeking – especially in relation to women’s agency and power in care-seeking – it is unknown if it is literacy itself that brings about an increase in women’s agency and power. We lacked information on how the literate women without schooling in our sample acquired their skills (in fact, the DHS dataset omitted the survey’s single question on whether women had participated in literacy programs outside of primary school). Women with little or no formal schooling who still manage to learn to read may be different from other women in certain ways. It might be those differences that explain and contribute to greater care-seeking agency and sense of empowerment. Further, actual care-seeking was measured only for women who had experienced STIs or symptoms of STIs. Sexual health is a sensitive topic in Nepal, and those women willing to tell DHS surveyors of problems related to STIs might be different than women in general. For example, regardless of being literate or not, these women might have certain characteristics that would make them both more likely to report STIs, as well as seek care for STIs, thereby potentially confounding the associations between literacy and care-seeking.

Finally, we acknowledge that the DHS uses a simplistic method for assessing literacy, which fails to take into account the multiple types of literacy that have been identified and the fact that literacy skills lie along a continuous spectrum [19,27]. More reliable and nuanced methods exist to assess literacy, such as those used by the UNICEF Nepal Literacy and Health Survey [15]. However, while we recognize these deficiencies and hope that improved methods will be adopted for the DHS in the future, the DHS dataset is valuable given that it contains a large, nationally representative sample. Moreover, because all DHS surveys generally incorporate the same questions, our analysis allows for the possibility of comparisons across surveys.

## Conclusions

Our findings provide support for increased implementation of adult literacy programs for women with little or no formal schooling. At the same time, we recommend further research using better literacy assessment methods than those of the DHS to confirm the associations we have observed, to help clarify the relationships among literacy, education, and healthcare-seeking, and to examine the different ways in which women acquire literacy skills outside of the formal education system. In particular, longitudinal research establishing which aspects and types of literacy improve health outcomes among women of low educational attainment could motivate more tailored programs, which may be especially important for women unable to return to formal schooling.

## Competing interests

The authors declare that they have no competing interests.

## Authors’ contributions

YL contributed to the study concept and design, literature review, statistical analysis and interpretation of data, and preparation of the manuscript. EB contributed to the literature review, data interpretation, and preparation of the manuscript. PS contributed to the study concept and design and oversaw the study. All three authors revised the manuscript for important intellectual content and approved the final manuscript.
